# Plyometrics with reactive agility or isometrics: divergent effects on change-of-direction ability, sprint, and strength adaptations in adolescent female handball players

**DOI:** 10.3389/fbioe.2026.1765655

**Published:** 2026-06-17

**Authors:** Souad Rebhi, Yosser Cherni, Mehréz Hammami, Dustin J. Oranchuk, René Schwesig, Mohamed Souhaiel Chelly

**Affiliations:** 1 Research Laboratory (LR23JS01) “Sport Performance, Health and Society”, Higher Institute of Sport and Physical Education of Ksar Saîd, University of Manouba, Tunis, Tunisia; 2 Higher Institute of Sport and Physical Education of Ksar Said, University of Manouba, Tunis, Tunisia; 3 Department of Human Physiology and Nutrition, College of Nursing and Health Sciences, University of Colorado Colorado Springs, Colorado Springs, CO, United States; 4 Muscle Morphology, Mechanics, and Performance Laboratory, Department of Physical Medicine and Rehabilitation, University of Colorado Anschutz Medical Campus, Aurora, CO, United States; 5 Department of Orthopedic and Trauma Surgery, Martin-Luther-University Halle-Wittenberg, University Medicine, Halle, Germany

**Keywords:** muscle performance, neuromuscular training, resistance workout, speed, team sports

## Abstract

**Introduction:**

Plyometric training, reactive agility, and isometric training can improve lower limb muscle performance, although the effectiveness of their combination remains unknown in adolescent female handball players. This study compared the effects of 8 weeks of plyometric training combined with either reactive agility or isometrics.

**Methods:**

Female participants were randomly assigned to control (CONT; *n* = 12; 14.1 ± 0.4 years), Combined Plyometrics and Reactive Agility Training (CPRAT); *n* = 12; 13.9 ± 0.5 years), or Combined Plyometrics and Isometric Training (CPIT); *n* = 12; 13.9 ± 0.5 years) groups. The intervention groups trained twice/week for 8 weeks. Performance was assessed before and after the interventions using: 10 m sprint performance, Repeated-Change-Of-Direction (RCOD), Y-shaped agility, T-half, throwing velocity, countermovement jump (CMJ), maximal isometric force and rate of force development (RFD) of knee extension muscle tests. Upper and lower limb muscle volume was estimated anthropometrically. A 3 × 2 mixed analysis of variance was employed to examine the interaction effects (group x time).

**Results:**

CPRAT led to significantly greater RCOD improvements (p < 0.01) and T-half performance (p < 0.001). No significant difference was observed between CPIT vs. CONT. In contrast, (CPIT) showed significantly greater improvements in lower limb muscle volume and knee extension maximal isometric force (p < 0.05) than CPRAT or CONT. Results between experimental groups remained statistically unchanged for Y-Shaped Test, RFD, and lower and upper limb muscle volume. Results for both experimental groups remained statistically unchanged compared to CONT for 10 m sprints, throwing velocity, and CMJ height.

**Discussion:**

The CPRAT protocol is superior for developing multidirectional and reactive agility. The CPIT protocol maximizes isometric strength but has limited transfer to high-velocity dynamic tasks. The CPRAT procedure should be selected when the main purpose is to boost agility and COD. However, when developing lower-limb maximal strength is the primary goal, the CPIT treatment should be applied.

## Introduction

Handball is categorized as a high-intensity, intermittent sport (Chelly et al., 2011; Manchado et al., 2013; Luteberget and Spencer, 2017; Luteberget et al., 2018). Handball players must therefore frequently perform high-intensity exercises like sprinting, jumping, throwing, and rapid directional changes (Karcher and Buchheit, 2014; Michalsik et al., 2015). They also need to make physical contact to provide the throwing player with positional advantages during team-handball specific movement (Wagner et al., 2014). Power, strength, agility, and postural stability are all essential for these actions (Bayios et al., 2001; Ortega and Lavie, 2018). Sex-related differences in neuromuscular characteristics should also be considered. Female athletes typically exhibit lower maximal strength and rate of force development (Hannah et al., 2012), alongside differences in neuromuscular control, which may influence performance and training adaptations (Hewett et al., 2005; Hunter, 2014). Therefore, it is crucial to develop effective training methods to improve handball players’ physical performance attributes and optimize athletic performance.

The application of plyometric training methods is common (Wagner et al., 2014) because it provides the required stimuli to enhance high-velocity performance in both pubertal and prepubertal populations (Matavulj et al., 2001; Dello Iacono et al., 2017; Hammami et al., 2019b; Chaabene et al., 2021). Such a regimen is natural to many sports that emphasize jumping, throwing, hopping, and skipping, and is particularly appropriate when developing movements like vertical jumping, as in handball players (Dello Iacono et al., 2017; Hammami et al., 2019b; Chaabene et al., 2021). While plyometric training has been studied extensively in various athletic populations, few researches has focused specifically on its effects combined with reactive agility or isometric exercise on adolescent female handball players. Understanding the potential benefits and differences between plyometric training combined with either reactive agility or isometric training in this population is crucial for designing evidence-based training programs that optimize their athletic development.

Several investigations and reviews have confirmed the effectiveness of plyometric training in improving the physical performance of athletes across disciplines, including basketball (Cherni et al., 2020; Ramirez-Campillo et al., 2022), soccer (Bedoya et al., 2015), and volleyball (Markovic, 2007; Ramirez-Campillo et al., 2021). Meanwhile, a growing body of literature examines the effects of plyometric training on the physical performance of handball players (Hammami et al., 2019a; Aloui et al., 2021). However, there is no comprehensive summary of this evidence. Isometric training may be particularly relevant for female athletes, as it can enhance maximal force production and neuromuscular function without high movement velocities (Oranchuk et al., 2019), which may help address commonly reported deficits in strength and rate of force development (Hannah et al., 2012). In addition, numerous scientific studies have demonstrated the effectiveness of combined training (plyometrics and isometrics). The combined approach yielded greater improvements in sprinting, 3-step running throw, running throw, and jumping performance than the contrast strength training group (Allégue et al., 2023).

Technical (dribbling, shooting, passing) and tactical skills are important factors that determine an athlete’s and the team’s success (Burgess and Naughton, 2010). Open-skill environments create unpredictable situations in which players must make quick decisions and respond appropriately to randomly occurring external stimuli (Di Russo et al., 2010). This response is often associated with body movement, which is called reactive agility (Sekulic et al., 2014; Spasic et al., 2015).

Reactive agility is considered one of the most important factors determining team performance in team sports, racquet sports, and combat sports (Lockie et al., 2014; Scanlan et al., 2014; Spasic et al., 2015). It could be considered as a critical pillar of sports performance by integrating motor, technical, and perceptual-cognitive factors, offering superior ecological validity compared to traditional agility tests (Horníková et al., 2021). It includes perceptual and decision-making processes (cognitive factors), physical factors (such as linear and Change-Of-Direction (COD) speed, leg muscle qualities), and, in some cases, technical factors (Sheppard and Young, 2006). This may be especially important in female players, where improved perceptual-motor integration could enhance movement efficiency (Hewett et al., 2005) and compensate for lower force production capacities (Hannah et al., 2012). Several reactive agility tests include sport-specific stimuli, mainly visual light stimuli (Sekulic et al., 2013; Pojskic et al., 2018). These tests may differentiate between higher- and lower-skilled players and players of different ages (Pojskic et al., 2018). Furthermore, current evidence highlights that response-based tasks to unpredictable stimuli are more sensitive in identifying high-level athletic proficiency (Chow et al., 2022).

Although plyometric training has been shown to be beneficial, it is still unclear if combining it with reactive agility exercises, which target perceptual-cognitive coupling, or isometric exercises, which concentrate on force production, is more beneficial for the unique needs of female adolescent handball players. To decide whether training should focus on maximal force production or decision-making speed during this crucial developmental stage, it is necessary to compare these two different paradigms. Thus, by determining the most time-efficient and sport-specific combination for optimizing performance in female adolescent handball players, our study closes a major gap in the literature.

Therefore, the purpose of the present study was to compare the effects of 8 weeks of combined plyometric and reactive agility training (CPRAT) versus combined plyometric and isometric training (CPIT) on sprinting, change of direction (COD), throwing, and strength. We hypothesized that both programs would substantially improve performance (COD, sprint, strength and reactive agility) compared to the control group.

## Materials and methods

### Experimental design

The study examined the effect of an 8-week combined plyometric training program (CPRAT vs CPIT) on physical performance in junior female handball players. The training interventions were conducted during the in-season period of 2023–2024. In the week before the intervention, two 80–90-min sessions familiarized players with all test procedures. Initial and final test measurements were made at the same time of day (17:00–19:00 h), under approximately the same environmental conditions (temperature: 16 °C–20 °C), at least 3 days after the most recent competition, and five to 9 days after the last training session. Measurements were made in a fixed order over 5 days, immediately before and 4 days after the last strength training session. Participants did not engage in any strenuous exercise for 24 h before testing, and no food or caffeine-containing drinks were consumed for 2 hours before testing. A standardized warm-up (10–20 min of low to moderate-intensity aerobic exercise and dynamic stretching) preceded all tests. The subjects were carefully familiarized with the techniques of circuit training and lifting for 2 weeks before measurements and training began. They were also familiarized with plyometric, isometric, and reactive agility training.

The Institute’s Committee on Research approved all procedures for the Medical Sciences (Manouba University Ethics Committee: LR23JS01). The study was conducted in accordance with the latest version of the Declaration of Helsinki. Written informed parental consent (for those <18 years) and participants’ assent were obtained before the start of the study. All participants and their parents/legal representatives were fully informed about the experimental protocol and potential risks and benefits. After giving their written content to be part of the investigation, the handball players were accepted and included in the study group. Moreover, the players were motivated to give maximal effort with strong verbal encouragement during all tests.

### Participants

Thirty-six adolescent female handball players participated in this study. They had at least 5 years of handball experience. All the players had some experience with plyometric training, but did not perform it weekly. They were examined by the team physician, with a particular focus on conditions that might preclude plyometric training, and all were found to be in good health. Players were divided by playing position, and players from each position were then randomly assigned to the plyometric and reactive agility training group (CPRAT; *n* = 12), plyometric and isometric strength group (CPIT; *n* = 12), or control group (CONT; *n* = 12). Anthropometric characteristics of both experimental groups and the control group are provided in Table 1.

**TABLE 1:** Physical characteristics of participants.

Group	Age [years]	Weight [kg]	Height [cm]	Body fat [%]	PHV [years]
CPRAT	13.9 ± 0.5	61.1 ± 10.9	167 ± 3	12.6 ± 0.3	1.6 ± 0.3
CPIT	13.9 ± 0.5	59.7 ± 9.4	166 ± 3	12.3 ± 1.5	1.6 ± 0.4
CONT	14.1 ± 0.4	65.2 ± 6.8	167 ± 5	12.0 ± 1.3	1.6 ± 0.4

CPRAT: combined plyometric and reactive agility training; CPIT: combined plyometric and isometric training; CONT: control; PHV: peak height velocity.

All groups were trained for 2 months, and were 1 month into the competitive season before the intervention. All subjects had achieved good overall physical preparation at the beginning of the season (six training sessions per week for 6 weeks). During the competition season, all participants were involved in five to six training sessions per week (90–120 min/session) and one competitive match per week, with training focused on handball-specific tactical and technical skills, dynamic strength training, and aerobic training (on- and off-court). Each Tuesday and Thursday for 8 weeks, the experimental groups replaced a part of their standard regimen with the plyometric training program. Both experimental groups (i.e., CPRAT and CPIT) performed their programs in place of some handball-specific drills, so overall training time was similar across groups. Participants who missed more than 10% of the total training sessions and/or more than two consecutive sessions were excluded from the study.

### Testing procedures

A standardized warm-up (10–20 min of low to moderate-intensity aerobic exercise and dynamic stretching) preceded all tests. All tests were separated by a five to 10-min break. Each player participated in a familiarization trial and two test trials. The best out of two trials was registered for further analyses. All tests were performed on a wooden surface at the same time of day.

Measurements were made in a fixed order over 5 days, immediately before and 4 days after the last plyometric training session. On the first test day, anthropometry measures, were conducted, followed by a 10 m sprint; three trials were allowed for this test (separated by six–eight minutes of recovery), and the best performance times were noted using paired photocells (Microgate, Bolzano, Italy). The second day was devoted to the Y-Shaped Test 10 m (Oliver and Meyers, 2009), followed by a ball-throwing velocity test. On the third day, countermovement jump was conducted. On the fourth day, two changes of direction tests (T-half agility test (T-half; Sassi et al., 2009)) and Repeated Change-Of-Direction test (RCOD) (Wong del et al., 2012) were performed. On the fifth day, the maximal isometric forces were assessed using the “Kinvent” device (Hahn, 2011; Johnston et al., 2022).

### Day 1

#### Anthropometry

Anthropometric measurements included standing and sitting body height (stadiometer accuracy of 0.1 cm; Holtain, Crosswell, Crymych, Pembs, United Kingdom) and body mass (0.1 kg; Tanita BF683W scales, Munich, Germany). The overall percentage of body fat was estimated from the biceps, triceps, subscapular, and suprailiac skinfolds, using the equations for children and adolescent females (Durnin and Womersley, 1974):

$$\%\;Body\;fat=\frac{495}{D}-450\;where\;D=1.1369-0.0598\;\left(\log\sum 4\;Skinfolds\right)$$

Maturity offset status was calculated from peak height velocity (Mirwald et al., 2002):

$$\text{Maturity offset}=-9.38+\left(0.000188\;\times \text{leg length}\times \text{ sitting height}\right)+\left(0.0022\;\times \text{age}\times \text{ leg length}\right)+\left(0.00584\;\times \text{age}\times \text{ sitting height}\right)+\left(0.0769\;\times \text{weight}/\text{height ratio}\right)$$

Circumferences and skin-fold thickness at different levels of the thigh and the calf, the arm and the forearm, the length of the lower and upper limbs, and the breadth of the humeral and femoral condyles were measured to estimate the muscle volume of the upper and lower limbs, as described previously (Shephard et al., 1988).

#### Sprint performance

Players started from a split stance standing position, with the front foot 0.2 m from the first photocell beam (Microgate, SARL, Bolzano, Italy) and sprinted for 15 m on command. Split times for 10 m distance was recorded for analysis (Cherni et al., 2020). The intra-class correlation coefficients (ICC) and the coefficients of variation (CV) values were 0.89% and 4.4% respectively.

### Day 2

#### Y-shaped agility test

The reactive Y-Shaped agility test evaluates the reactive agility. The task was to run as fast as possible 5 m forward, then to change direction at a 45° angle to the right or left, as pre-planned by the light timing system Witty Sem (Microgate Srl, Bolzano, Italy), and run another 5 m to one of two finish gates. Participants were asked not to initiate the change of direction until they had passed through the middle gate, and the change of direction was performed as a reaction to a visual stimulus, one of two finish gates lit up green. Participants were instructed not to predict which gate would light up. They started 0.3 m behind the first pair of photocells, which were set at a width of 1.5 m, and height between 1 and 1.2 m (adapted to the height of participants). Two trials to the left and right were taken, and the best of them was used for further analysis. This test was found to be reliable (Oliver and Meyers, 2009).

#### Ball throwing velocity tests

One type of overarm throw (Chelly et al., 2014) was performed on an indoor handball court: the jump shot. The ball throwing velocity was measured using a radar Stalker ATS II system™ (Radar Sales, Minneapolis, MN, USA), hand-held at shoulder level. The maximal ball velocity was noted for three consecutive trials for each throw type, each separated by at least 15 s of recovery. Players were immediately informed of their performance to maximize motivation, and the fastest of their three values was recorded. The reproducibility of the ball throwing velocity has been verified (ICC = 0.97; CV = 21%).

### Day 3

#### Vertical jumps

The Optojump System (Microgate SARL, Bolzano, Italy) was used to measure jump height. Under the researcher’s supervision, the countermovement jump (CMJ) began with the individuals standing straight up, quickly bending their knees to 90°, then pushing off (Allégue et al., 2023). The reproductibility of the vertical jump test has been verified (ICC = 0.97; CV = 11.5%).

### Day 4

#### T-half test

The T-Half test determined speed with directional changes (forward sprinting, left and right shuffling, and backward running). Subjects began with both feet behind the starting line (A). They sprinted forward for 5 m distance to cone B, touching its base with the right hand. Facing forward and without crossing the feet, they next shuffled leftward to cone C covering a distance of 2.5 m, touching its base with the left hand. They then performed a lateral shuffle to the right toward cone D, positioned 5 m away, touching its base with the right hand. They next shuffled leftward to cone B, touching its base, and finally, ran backward to line A (Sassi et al., 2009). Anyone who crossed one foot in front of the other, failed to touch the base of a cone, and/or failed to face forward throughout had to repeat the test. Performance times were recorded by paired photocells (Microgate, Bolzano, Italy). The reproductibility of the T-Half Test has been verified (ICC = 0.98; CV = 10%).

#### Repeated change-of-direction test (RCOD)

The RCOD consisted of six 20 m sprints with 25 s active recovery intervals. Each sprint consisted of four 100° COD maneuvers at every 4 m as shown (Beckett et al., 2009). During the active recovery, the subjects jogged slowly back to the starting line and waited for the next sprint. The sprint time for 20 m was measured using an infrared timing system (Microgate, Bolzano, Italy) located at the starting and finishing lines, 1 m above the ground. Each player was verbally encouraged to give maximal effort during all RCOD tests. The best time in a single trial (RCOD-Best), and the total time for the six sprints repetitions (RCOD-Total time) were notes, and RCOD-decrement was calculated according to the formula (Glaister et al., 2008):

$$\text{RCOD}-\text{decrement}=100\;x\;\left(\frac{Total\;sprint\;time}{ideal\;sprint\;time}\right)-100$$

where ideal sprint time = number of sprint x RCOD-Best.

#### Maximal voluntary isometric contraction force test (MVIC)

The MVIC is an isometric test which assesses the maximum neuromuscular capacity of the knee extensors. This test is used to determine deficits in strength or rate of force development. A traction dynamometer (Kinvent, K-PULL, Montpellier, France) was used. The link was fixed in place. For lower-limb measurements, the participant sat on the edge of a table with their feet not touching the floor. Their knees were bent at 45°. Then the strap was put around the participant’s ankle, and they were asked to push forward as hard as possible (Hahn, 2011; Johnston et al., 2022). The measured parameter was Rate of Force Development (RFD) of the left limb. Each participant was allowed three trials, each separated by a 1-min recovery period. The highest value from the three trials was used.

### Training programs

#### The combined plyometric and reactive agility training program

**FIGURE 1:**
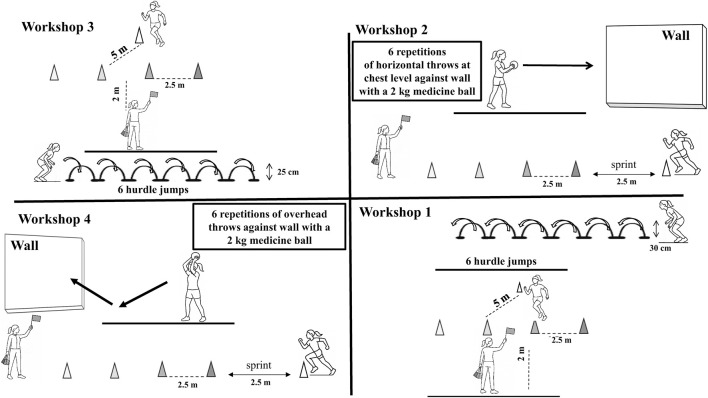
The combined plyometric and reactive agility training program.

This combined training program (Figure 1) took the form of circuit training divided into four main workshops. Participants should complete one repetition at each workshop and then move on to the next workshop without any recovery period. The participants were instructed to perform a reactive agility and a plyometric exercise. The completion of the four successive workshops is counted as one set. Each participant should complete four sets separated by 120 s of passive recovery time.

Workshop 1: (a) Four cones of different colours (green, red, blue and yellow) were placed 2.5 m apart. A white cone was placed in front of the other cones at a distance of 5 m (the starting cone). The coach indicated the movement towards the cones chosen at random using flags of the same colours as the cones. At the signal, the participant had to run as quickly as possible forward and touch the indicated cone, then turn and touch the white cone, (b) six repeated jumps over 30 cm hurdles.

Workshop 2: (a) Four cones of different colours (green, red, blue and yellow) were placed in a straight line 2.5 m apart. The starting cone was white and placed before the other cones on the same line. The coach indicated the movement towards the cones chosen at random using flags of the same colours as the cones. At the signal, the participant had to run as quickly as possible towards the cone indicated and then run back towards the starting cone, (b) six horizontal throws of a 2 kg medicine ball located at chest level toward the wall.

Workshop 3: (a) Four cones of different colours (green, red, blue and yellow) were placed 2.5 m apart. A white cone was placed in front of the other cones at a distance of 5 m (the starting cone). The coach indicated the movement towards the cones chosen at random using flags of the same colours as the cones. At the signal, the participant had to run as quickly as possible forward and touch the indicated cone, then turn and touch the white cone, (b) six hurdle steps over hurdles 25 cm in height (three on each leg).

Workshop 4: (a) Four cones of different colours (green, red, blue and yellow) were placed in a straight line 2.5 m apart. The starting cone was white and placed before the other cones on the same line. The coach indicated the movement towards the cones chosen at random using flags of the same colours as the cones. At the signal, the participant had to run as quickly as possible towards the cone indicated and then run back towards the starting cone, (b) throw in with a 2 kg medicine ball.

#### The combined plyometric and isometric training program

**FIGURE 2:**
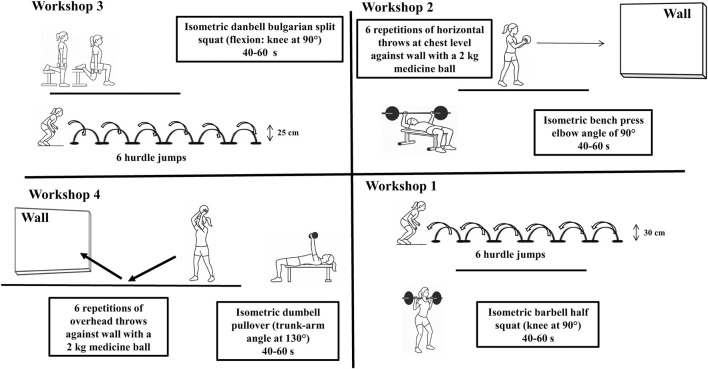
The combined plyometric and isometric training program.

All participants in this group underwent a technical training session on the bench press and pull-over exercises, which were used for their isometric training (Figure 2). The one-repetition maximum value (1-RM) was also determined as described in the scientific literature (Hermassi et al., 2010). The combined plyometric and isometric training program takes the form of circuit training divided into four main workshops. The participants should complete one repetition at each workshop and then move on to the next workshop without any recovery period. The participants were instructed to perform an isometric and a plyometric exercise. The completion of the four successive workshops is counted as one set. Each participant should complete four sets separated by 120 s of passive recovery time.

Workshop 1: (a) Isometric barbell half squat with a knee angle of 90°. For the first 4 weeks, the participant maintained an isometric hold for 40 s per exercise. The isometric duration progressively increased by 5 s each week up to 60 s, (b) six repeated jumps over 30 cm hurdles.

Workshop 2: (a) Isometric barbell bench press with an elbow angle of 90°, (b) six horizontal throws of a 2 kg medicine ball located at chest level toward the wall.

Workshop 3: (a) Isometric dumbbell Bulgarian split squat (leg on the bench and support leg with a knee angle of 90°), (b) six hurdle steps over hurdles 25 cm in height (three on each leg).

Workshop 4: (a) Isometric dumbbell pull-over with a trunk-arm angle of 130°, (b) throw in with a 2 kg medicine ball.

The CPIT program was divided into four stations. Each station included three exercises (four sets with one-two minutes of rest). The participants were instructed to perform an isometric, a plyometric, and a speed exercise, which could be either a sprint or a throw, depending on the session. The stations were completed four times during each session, with all sets separated by 120 s of passive rest. The isometric exercise was followed immediately by a plyometric exercise. The quantification of workload is described in Table 2.

**TABLE 2:** The training program assigned for the isometric training group.

Cycle	Week	% 1RM	Set × contraction time [s]	Recovery time [min]
1	1	50	3 ×40	1–2
	2	55	3 × 40	1–2
	3	60	3 × 45	1–2
	4	60	3 × 45	1–2
2	5	65	3 × 50	1–2
	6	65	3 × 55	1–2
	7	65	3 × 60	1–2
	8	65	3 × 60	1–2

#### Training load

The rated perceived exertion (RPE) was used to quantify the internal load of the participant during training session. All the participants responded with regard to their perception on a 0-to-10-point scale (Foster et al., 2001), following 30 min of their first training session and their last training session (session 16). During the first training session, RPE values were 7.92 ± 0.90, 7.33 ± 0.65, and 7.58 ± 1.08 for the CPRAT, CPIT, and CONT groups, respectively. During the last training session, RPE values were 6.33 ± 0.78, 6.67 ± 0.65, and 6.42 ± 0.79 for the CPRAT, CPIT, and CONT groups, respectively. A one-way analysis of variance revealed no significant differences in RPE between the three groups during either the first or the last training session, confirming that the training load was equivalent across groups.

### Statistical analyses

All statistical analyses were performed using SPSS Statistics version 31.0 (IBM, Armonk, NY, USA).

Before inferential testing, data normality was assessed using Shapiro-Wilk Tests and visual inspection of Q-Q plots. Homogeneity of variances was verified for all dependent variables using Levene’s test.

A priori a sample size calculation (nQuery Advisor 4.0; Statistical Solutions, Saugus, MA, USA) was performed to determine the appropriate sample size (primary outcome: CMJ). The calculation based on the results of Bouteraa et al. (2020) and the following assumptions: a two-sided hypothesis, alpha-level: p < 0.05, power 1-β: 0.8, mean difference (SD) intervention groups: 28.8 (3.5) cm, mean difference (SD) control group: 24.4 (3.5) cm. Based on the calculated effect size from 0.58, the analysis revealed that 11 athletes per group were necessary to detect relevant differences between groups over the time. Assuming a dropout rate of 20% (*n* = 7), 40 athletes should initially be recruited.

A 3 × 2 mixed analysis of variance (ANOVA) was employed to examine the interaction effects (group x time). Baseline differences between groups were analysed using a one-way ANOVA. When significant inter-group baseline differences were identified that could confound the results, an analysis of covariance (ANCOVA) was performed on the change scores to control for these initial disparities. For variables that did not meet the assumptions of normality, nonparametric alternatives, including the Kruskal-Wallis test and Quade’s ANCOVA, were applied.

Effect sizes were calculated for all parameters. For ANOVA and ANCOVA procedures, partial eta-squared (η_p_^2^) was used, with interpretations as follows: small (0.01–0.06), medium (0.06–0.14), and large (>0.14; Hopkins et al., 2009). Cohen’s d was calculated and interpreted according to Hopkins’ recommendations: trivial (0.00–0.19), small (0.20–0.59), moderate (0.60–1.19), large (1.20–1.99), and very large (≥2.00; Hopkins et al., 2009).

Test-retest reliability was determined using intraclass correlation coefficients (ICC) with 95% confidence intervals, and coefficients of variation (CV%). CV% was calculated as: CV% = (standard deviation/mean) × 100, following established guidelines for sports performance assessment (Vincent, 1995; Schabort et al., 1998). The level of significance for all analyses was set a priori at p < 0.05 and η_p_^2^ > 0.15 and d > 0.80.

## Results

After the intervention, both experimental groups showed significant decreases in times for RCOD-Best, RCOD-TT and Y- Shaped Test 10 m relative to controls, with no significant-relevant intergroup differences in response between trained groups.

The muscle volumes of the upper and lower limbs, increased significantly in all three groups (Table 3). The CPIT group appeared to have made the most progress, as the ANOVA detected a significant interaction effect (p < 0.05) in its favour compared, with CONT showing lower total lower limb muscle volume. However, the muscle volume in the CPRAT group remained stable, with no significant change. On the other hand, the two combined training programs in this study did not induce any change in upper limb muscle volume, as no significant group × time interaction was detected.

**TABLE 3:** Lower and upper limbs muscle volume in the three groups before and after the 8-week intervention (*n* = 12 for each group). ES = effect size: Significance and relevance threshold: d > 0.5 and p < 0.05 and η_p_^2^ > 0.15. Relevant effects marked in bold.

Variables	Group	Pre	Post	ES	Group		Group x time		Post-hoc-analysis
				d	p	η_p_^2^	p	η_p_^2^	p
Lower limb muscle volume [L]	CPRAT	7.36 ± 1.18	8.07 ± 1.26	**0.58**	**0.003**	**0.16**	0.996	0	CPRAT vs. CPIT: 0.070 CPIT vs. CONT: **0.003**
	CPIT	8.42 ± 2.21	9.21 ± 2.37	0.35					
	CONT	6.81 ± 1.12	7.56 ± 1.14	**0.66**					
Upper limb muscle volume [L]	CPRAT	2.18 ± 0.66	2.49 ± 0.68	0.47	0.694	0.01	0.938	0	**-**
	CPIT	2.32 ± 0.89	2.67 ± 0.95	0.38					
	CONT	2.22 ± 0.72	2.42 ± 0.68	0.29					

CPRAT: combined plyometric and reactive agility training program; CPIT: combined plyometric and isometric training program; CONT: control group; ES: Effect size. The control group showed the largest improvement regarding lower limb muscle volume. The effect is almost twice as large as in CPIT, group (0.35 vs. 0.66) and this difference is clinically relevant (p = 0.003).

Squat Jump, CMJ, throwing velocity, and 10 m sprinting performances remained unchanged after the 8-week intervention (Table 4). On the other hand, and in contrast to the CPIT group, the CPRAT group showed a substantial enhancement in RCOD-Best and RCOD-TT compared to CONT (p < 0.01 for all measurements; Table 5).

**TABLE 4:** Vertical jump, throwing velocity and sprinting performances in the three groups before and after the 8-week intervention (*n* = 12 for each group).

Variables	Group	Pre	Post	ES	Group		Group x time		Post-hoc-analysis
				d	p	η_p_^2^	p	η_p_^2^	p
CMJ [cm]	CPRAT	29.7 ± 4.57	33.1 ± 4.24	**0.77**	0.555	0.02	0.633	0.01	**-**
	CPIT	32.1 ± 3.93	33.4 ± 4.03	**0.58**					
	CONT	31.4 ± 4.26	32.8 ± 4.31	0.33					
10 m [s]	CPRAT	2.71 ± 0.13	2.66 ± 0.12	0.40	0.179	0.05	0.945	0	-
	CPIT	2.67 ± 0.09	2.63 ± 0.09	0.44					
	CONT	2.73 ± 0.13	2.69 ± 0.11	0.33					
Ball throwing velocity [m/s]	CPRAT	50.8 ± 12.4	60.3 ± 13.3	0.74	0.344	0.03	0.696	0.01	**-**
	CPIT	51.1 ± 9.21	56.5 ± 10.6	0.55					
	CONT	56.1 ± 8.91	60.7 ± 10.1	0.48					

CPRAT: combined plyometric and reactive agility training program; CPIT: combined plyometric and isometric training program; CONT: control group; ES: Effect size. Significance and relevance threshold: d > 0.5 and p < 0.05 and η_p_^2^ > 0.15. Relevant effects marked in bold. Regarding CMJ, the intervention effects in the experimental groups were markedly larger than in the control group. The effect in the CPRAT, group was more than twice as large as in the control group (0.33 vs. 0.77).

**TABLE 5:** Repeated Change-of-direction performance in the three groups before and after the 8-week intervention (*n* = 12 for each group).

Variable	Group	Pre	Post	ES	Group		Group x time		Post-hoc-analysis
				d	p	η_p_^2^	p	η_p_^2^	p
RCOD-Best [s]	CPRAT	7.33 ± 0.61	7.23 ± 0.61	0.16	**0.003**	**0.16**	0.991	0	CPRAT vs. CONT: **0.002** CPRAT vs. CPIT: 0.065
	CPIT	7.74 ± 0.56	7.67 ± 0.55	0.13					
	CONT	7.95 ± 0.71	7.89 ± 0.72	0.08					
RCOD-TT [s]	CPRAT	44.2 ± 3.69	43.6 ± 3.64	0.16	**0.003**	**0.16**	0.996	0	CPRAT vs. CONT: **0.002** CPRAT vs. CPIT: **0.066**
	CPIT	46.7 ± 3.37	46.2 ± 3.25	0.15					
	CONT	48.0 ± 4.33	47.5 ± 4.16	0.12					
RCOD-decrement [s]	CPRAT	0.52 ± 0.21	0.54 ± 0.28	0.08	0.329	0.07	0.594	0.03	CPRAT vs. CONT: 0.889 CPRAT vs. CPIT: 1.000
	CPIT	0.48 ± 0.18	0.44 ± 0.18	0.22					
	CONT	0.54 ± 0.24	0.90 ± 1.58	0.40					

CPRAT: combined plyometric and reactive agility training program; CPIT: combined plyometric and isometric training program; CONT: control group; RCOD: repeated change of direction; RCOD-Best: Best time in a single trial; RCOD-TT: total time for the six sprints repetitions; ES, effect size: Significance and relevance threshold: d > 0.5 and p < 0.05 and η_p_^2^ > 0.15. Relevant effects marked in bold. The CPRAT, group displayed a significantly (p = 0.002) larger improvement compared with the control group. However, both effects were very low (0.08 and 0.16).

The CPRAT group showed a large enhancement in the planned agility test (T-half test) compared to CONT (p < 0.001; Table 6), with no change in performance for CPIT. In addition, the CPRAT group showed a significant increase in the non-planned agility test (Y- Shaped Test 10 m), especially in the Y- Shaped Test 10 m. Moreover, the Y- Shaped Test 10 m for the CPIT group were significantly improved compared to CONT.

**TABLE 6:** T-half test and Y-Shaped test in the three groups after the 8-week intervention (*n* = 12 for each group).

Variables	Group	Pre	Post	ES	Group		Group x time		Post-hoc-analysis
				d	p	η_p_^2^	p	η_p_^2^	p
T-half test [s]	CPRAT	6.58 ± 0.66	6.07 ± 0.64	**0.79**	**<0.001**	**0.23**	0.433	0.03	CPRAT vs. CONT: < **0.001** CPRAT vs. CPIT: **0.035**
	CPIT	6.87 ± 0.56	6.78 ± 0.58	0.16					
	CONT	7.21 ± 0.77	7.13 ± 0.78	0.10					
Y-Shaped test 10 m [s]	CPRAT	3.19 ± 0.30	2.90 ± 0.27	**1.02**	**0.002**	**0.17**	0.408	0.03	CPRAT vs. CONT: **0.006** CPIT vs. CONT: **0.006**
	CPIT	3.11 ± 0.20	2.98 ± 0.19	**0.67**					
	CONT	3.33 ± 0.26	3.21 ± 0.24	0.48					

ES, effect size: Significance and relevance threshold: d > 0.5 and p < 0.05 and η_p_^2^ > 0.15. Relevant effects marked in bold. The largest difference between the control group and any intervention group was observed for the parameter t-half test. The CPRAT, group showed an eight times greater improvement compared to the control group (0.79 vs. 0.10; p < 0.001). The longitudinal changes in the CPIT, group were at approximately the same level as in the control group (0.16 vs. 0.10), which the difference to the CPRAT, group was also significant (p = 0.035). Concerning the Y-Shaped test, both intervention groups showed relevant changes over the time, especially the CPRAT, group (d = 1.02) and related to the control group (p = 0.006).

The three groups showed a substantial enhancement (p < 0.01) in maximal isometric force (Table 7). In contrast, the lower limb maximal isometric force parameters showed a significant interaction effect in favour of the CPIT group compared with the CPRAT group. Almost the same kinetics of results were obtained for RFD. Indeed, this last parameter showed a significant improvement following our training program, where the results showed a group × time interaction in favour of the CPRAT group (p = 0.0024) compared to CONT (Table 7).

**TABLE 7:** Maximal isometric force and rate of force development of knee extensor muscle in the three groups before and after the 8-week intervention (*n* = 12 for each group).

Variables	Group	Pre	Post	ES	Group		Group x time		Post-hoc-analysis
				d	p	η_p_^2^	p	η_p_^2^	p
Maximal isometric force of knee extensor muscle of left leg [kg]	CPRAT	25.1 ± 3.34	27.3 ± 4.05	**0.60**	**0.003**	**0.16**	0.721	0.01	CPRAT vs. CONT: 0.032 CPRAT vs. CPIT: 0.003
	CPIT	21.7 ± 3.12	24.2 ± 3.01	**0.82**					
	CONT	23.2 ± 3.12	24.2 ± 3.19	**0.32**					
RFD-LLL [kg/s]	CPRAT	21.3 ± 4.15	24.4 ± 4.15	**0.75**	0.026	0.10	0.806	0.01	CPRAT vs. CONT: 0.024
	CPIT	19.0 ± 4.16	22.2 ± 4.29	**0.76**					
	CONT	18.6 ± 4.25	20.3 ± 4.89	0.37					

ES, effect size: Significance and relevance threshold: d > 0.5 and p < 0.05 and η_p_^2^ > 0.15. Relevant effects marked in bold. CPRAT: combined plyometric and reactive agility training group; CONT: control group; CPIT: combined plyometric and isometric training; RFD: rate of force development; LLL: left lower limb; RLL: right lower limb; ES: effect size; RFD-LLL: Rate of force development of left lower limb. The effect sizes for both parameter and intervention groups moved consistently at a medium level (range: 0.60–0.82). The CPRAT, group displayed in both parameters significant differences to the control group (p = 0.032 and p = 0.024). The largest effect and difference to the control group was calculated for the CPIT, group (d = 0.82) based on the maximal isometric force of knee extensor muscle of left leg.

## Discussion

### Summary of the main findings

This study compared the effects of two types of training programs on the athletic performance of junior female handball players. The results show that CPRAT yielded greater improvements in RCOD, T-half test, Y- Shaped Test 10 m and maximal strength.

### Linear sprinting (10 m sprint performance)

Sprint performances for 10 m distance remained statistically unchanged after the current intervention in both experimental groups. These findings are in accordance with some previous investigations such as Hammami et al. (2019b) who found no gains in 5 m, 10 m and 20 m sprint performances after 9-week upper and lower limb plyometric training in U14 female handball players, or Markovic et al. (2007) who found no improvements in 20 m sprint times after 10-week plyometric training in male physical education students, and Herrero et al. (2006) who found no significant gains in 20 m sprint times after 4-week electromyostimulation training in men.

Recently (Allégue et al., 2023), found no improvement in sprint performance after 8 weeks of contrast-strength training in male junior handball players. However, several reports found significant increases in sprint performance over short distances after plyometric training such as Idrizovic et al. (2018) who found that 12 weeks of plyometric training enhanced 20 m sprint performance in female volleyball players aged 16.6 years, or Hammami et al. (2016) who found gains in 5 m, 10 m and 20 m performances after 8-weeks of lower limb plyometric training programme. These discrepancies may reflect, in part, differences in methodology (youth vs. young soccer players; elite vs. regional soccer players; length of study; the format of the plyometric exercises; the frequency, duration, and progression of training; and its timing relative to the playing season). Several studies and practical observations have reported no significant improvements in sprint performance, particularly among adolescent female athletes. This outcome may appear counterintuitive, but can be explained by multiple physiological, developmental, and training-specific factors. Sprinting, especially over short distances like 10 m, demands maximal linear acceleration and horizontal force production.

However, plyometric exercises (e.g., vertical jumps) and reactive agility drills tend to emphasize vertical power and multidirectional movement patterns, which might not directly transfer to horizontal sprint performance. In this line, Spiteri et al. (2014) emphasized that the specificity of training is critical, and agility-based drills may not effectively carry over to linear sprinting unless specifically designed for that outcome. Another common reason for a lack of improvement is that the volume, frequency, or duration of the training program might be insufficient to induce measurable adaptations in sprinting. In addition, initial acceleration in short distance such as over 5 and 10 m has proven more difficult to enhance than maximal velocity, probably because of the smaller margin for improvement and the different forces involved (Christou et al., 2006; Kotzamanidis, 2006; Meylan and Malatesta, 2009). Likewise, combining two different training modalities such as reactive agility (cognitive and decision-based) and plyometrics (explosive neuromuscular), may lead to interference effects, particularly in younger or less experienced athletes. The body may struggle to adapt efficiently to both cognitive-motor and neuromuscular demands simultaneously.

In this line, Faigenbaum et al. (2009) noted that in youth populations, overloading the neuromuscular system with complex stimuli can lead to plateaued or negligible gains, particularly when exercises lack proper technical coaching or progression. Also, adolescent females undergo hormonal fluctuations that affect strength, coordination, and body composition. These biological changes can impair training responsiveness, especially during high-speed activities such as sprinting. Indeed, increased estrogen levels during puberty can influence ligament laxity, joint stability, and neuromuscular control, potentially reducing sprint efficiency. In addition, adolescent females may develop increased fat mass, which can negatively affect sprinting performance despite improvements in power or agility (Myer et al., 2009).

It is important to note that the explanations proposed above pertain exclusively to performance in short-distance accelerations, such as the 10 m sprint, and cannot be extrapolated to other phases of sprinting, notably the maximal velocity phase, which typically emerges at distances of 30 m or beyond.

Sprinting performance is a key factor in handball, especially in high-speed transitions and defensive recovery. While both isometric and plyometric training are commonly used to enhance strength and explosiveness, research and practice show that combining these modalities does not always lead to significant improvements in sprinting, particularly in female handball players. Sprinting demands a high rate of force development (RFD) in a very short ground contact time (around 100 ms during maximum velocity).

### Change-of-direction (T-half test; RCOD; Y-shaped test 10 m)

The CPRAT group showed a large enhancement in the planned agility test (T-half test) compared to CONT (p < 0.001; Table 6), with no change in performance for CPIT. Group × time interaction effects showed that the CPRAT group exhibited a substantial enhancement in RCOD-Best and RCOD-TT compared to CONT (p < 0.01 for both measurements), contrary to the CPIT group (Table 6), which showed unchanged statistical results compared to CONT. In addition, the CPRAT group showed a significant increase in the non-planned agility test, especially in Y-Shaped Test 10 m. However, the measured Y-Shaped Test 10 m performances was significantly improved in the CPIT group compared to CONT. Miller et al. (2006) found that a plyometric program also increased the Illinois Agility test scores of five girls and nine males (4.9% for the Illinois Agility test and 2.9% for the T-test).

Asadi and Arazi (2012) found that young male basketball players aged 19–20 years had 9% shorter T-test agility times after 6 weeks of high-intensity plyometric training (2 days/week). The T-test results of top male basketball players following 6 weeks of plyometric training demonstrated increased nerve conduction velocity, a shorter time needed for voluntary muscle activation, and improved coordination between the central nervous system signal and proprioceptive input as examples of neural adaptations that contribute to gains in the capacity to change direction quickly (Craig, 2004). Improved motor unit recruitment strategy (Miller et al., 2006; Asadi and Arazi, 2012; Asadi, 2013), improved inter-muscular coordination (Slimani et al., 2016), directional changes, anticipation, and decision-making processes are all fundamental perceptual components.

These findings suggest that a promising method for enhancing RCOD in adolescent female handball players is to alternate between plyometric and reactive agility training in the same session. We anticipated that training including reactive and plyometric agility would lead to improvements in performance on handball-related activities, such as sprinting, direction changes, and repeated direction changes.

Agility is a vital component of handball performance, requiring rapid changes in direction, acceleration, and deceleration, as well as decision-making during gameplay. In adolescent female handball players, improving agility can enhance performance in both offensive and defensive actions. While both CPRAT and CPIT may provide physical benefits, evidence suggests that CPRAT is more effective in improving agility performance. A combination of neuromuscular, biomechanical, and cognitive factors can explain this difference.

The CPRAT results could be supported by improvements in sensorimotor integration, reaction time, and neuromuscular coordination, all of which are essential for agile movements. It trains the central nervous system to produce fast, coordinated, and precise motor responses in unpredictable situations. In this line, Sheppard and Young (2006) highlighted that agility is not only a physical quality but also a perceptual and cognitive skill. Reactive agility training significantly improves this through stimulus-response drills that imitate game demands. Also, plyometric training enhances muscle power, reactive strength, and the efficiency of the stretch-shortening cycle. When combined with agility drills that emphasize cutting, braking, and turning mechanics, it leads to greater improvements in RCOD speed and movement efficiency. Indeed, adolescents benefit greatly from training that stimulates both neuromuscular and cognitive systems, as this enhances the efficiency of movement patterns under decision-making pressure.

In this line, Sheppard and Young (2006) found that reactive agility training improves cognitive agility, allowing athletes to better anticipate and respond to unpredictable scenarios during gameplay. Isometric exercises, however, do not involve dynamic eccentric-concentric muscle actions and thus offer limited benefit for RCOD performance. Furthermore, reactive agility drills include cognitive components such as perceptual processing, stimulus recognition, and decision-making, which are essential in real-match agility performance. In contrast, CPIT lacks any cognitive challenge, reducing its transferability to sport-specific agility. Conversely, isometric exercises improve maximum static strength at specific joint angles but do not involve movement, velocity, or direction changes. As a result, isometric training may fail to transfer effectively to agility performance. The CPIT primarily enhances static strength without affecting rate of force development or rapid directional change. Based on this premise, Tillin and Folland (2014) reported that isometric training improves muscle strength but has a limited effect on tasks involving speed and rapid movement, such as sprinting or agility drills.

Furthermore, the improvement in RCOD ability with plyometric training also reflected an increase in agility. These improvements in agility performance are consistent with previous meta-analyses examining athletes at different levels and stages of maturity (Asadi et al., 2017). Agility is primarily manifested through rapid change of direction, achieved by accelerating and decelerating the lower limbs in response to various situations or stimuli (Sheppard and Young, 2006). The decelerating phase mainly relies on the eccentric strength of the thigh muscles (Chaabene et al., 2018). In plyometric training, the greater inertia accumulated during the braking phase results in a greater eccentric load, which may enhance eccentric strength. The rapid switching between deceleration and acceleration primarily depends on neuromuscular adaptations, especially improved neural drive to the agonist muscles and enhanced inter- and intra-muscular coordination (Markovic and Mikulic, 2010).

### Maximal isometric force and rate of force development

The CPIT group showed a substantial increase in maximal force (p < 0.05), especially in the lower limbs (Table 7), relative to CONT and CPRAT. Both training strategies significantly improved most neuromuscular performance measures compared to the CONT. Allégue et al. (2023) indicated that alternating between isometric and plyometric exercises in the same session holds significant promise as a potential strategy to enhance explosive action in male handball players. In addition, training combining both moderate-intensity isometric and light-load, high-velocity plyometric drills would result in performance increments in handball-related tasks such as jumping, sprinting, and throwing.

The static nature of muscular contraction in isometric mode is often perceived as less beneficial for dynamic sports performance (Lum and Barbosa, 2019). Indeed, dynamic strength training is generally considered the preferred mode due to its ability to improve dynamic sports performance (Suchomel et al., 2018). Explosive actions, such as sprinting, are important in handball competition, and the gains observed after 8 weeks of CPRAT were substantial. These training benefits for muscle power may be attributed to structural and neuromuscular adaptations, such as an increase in the proportion of type IIx muscle fibers, enhanced tendon stiffness, improved motor unit recruitment, greater muscle coordination and enhanced reflex control (Radnor et al., 2018). This could be attributed to the specificity of the training, as plyometric training primarily focuses on vertical jumping, leading to greater improvements in vertical jump performance.

The research by Dello Iacono et al. (2017) confirmed this specificity. Furthermore, a specific pattern of training adaptations was noted in the 8-week training block. In fact, the CPIT group showed greater improvements than the CPRAT group in maximal voluntary force gain, underscoring the efficacy of isometric training for improving maximal voluntary force. In contrast, improvements in RFD relative to controls were observed in the CPRAT group, demonstrating the unique capacity of reactive agility training to enhance explosive force output. These dissociated outcomes aligned with the specificity principle, by showcasing how different supplemental training methods preferentially target distinct neuromuscular attributes; in this case, reactive agility training amplifies explosiveness, while isometric training centers on maximal strength. The morphological adaptations examined also aligned with these conclusions. Compared with the control group, the CPIT group showed greater increases in thigh and lower limb muscle volume, demonstrating that this training paradigm provides a significant hypertrophic stimulus. In contrast, the CPRAT group did not show significant improvements in any morphological characteristics compared with the control group.

These results suggest that, in the absence of significant muscle hypertrophy, the CPIT program produced notable structural adaptations, while the advantages of the CPRAT program seem to be mainly mediated by neural adaptations.

### Vertical jump

Vertical jump performance is a crucial athletic quality in handball, where players frequently engage in jumping actions for shooting, blocking, and rebounding. Enhancing vertical jump height reflects improvements in explosive lower-limb power, neuromuscular coordination, and effective use of the stretch-shortening cycle. The results of the current investigation showed that CPRAT and CPIT yielded large improvements in CMJ, but there were no differences between the two training regimens and the CONT. In accordance with our data, the replacement of a part of our standard handball training by an 8-week plyometric training program enhanced several characteristics of importance to handball performance, including the absolute and relative muscle power of the upper and lower limbs (p < 0.001), SJ and CMJ height, and power (Chelly et al., 2014). Plyometric training has been advocated for many years as a means of improving performance in sports where lower-body power is key to success. The improvements in vertical jump performance could be explained by an increase in fibre length (the number of sarcomeres in series (Vissing et al., 2008); or by the stress-related overload imposed on the body during plyometric training. The gains in vertical jumping are in line with Maio Alves et al. (2010), who found a 10% improvement in countermovement jumping in junior soccer players after 8 weeks of strength training.

Likewise, Franco-Márquez et al. (2015) found that adding 6 weeks of combined resistance and plyometric training to standard soccer training resulted in gains in countermovement jumping. While both CPRAT and CPIT offer benefits among adolescent female handball players, CPRAT appears more effective. This is due to its dynamic nature, better use of the stretch-shortening cycle, enhanced neuromuscular coordination, and cognitive engagement. On the other hand, isometric training may enhance strength at specific joint angles but lacks the movement specificity and velocity required to significantly improve vertical jumping ability (Sheppard and Young, 2006; Tillin and Folland, 2014; Hammami et al., 2019b).

## Limitations

Several limitations of this study should be acknowledged: First the undetermined effect of the menstrual cycle in female players, which may lead to significant variations in performance. Second, the study could have been strengthened by the use of EMG electrodes for better quantification of neuromuscular interactions and adaptations. Third, we did not evaluate the dominant leg, making it necessary to choose a test with the same number and the same angles of change of direction for left and right sides, to neutralize dominant leg effects. Fourth, individual differences in food habits and nutritional intake may have affected the training adaptations seen among participants because no controlled or uniform nutritional program was created or tracked during the experimental period. Lastly, the exclusive use of a 10 m sprint test to evaluate sprint ability could be viewed as a drawback because it primarily captures early acceleration capabilities and ignores other crucial sprinting periods, including 20 or 30 m lengths.

## Conclusion

This study clearly demonstrates, that in junior female handball players the specificity of adaptations generated by combining plyometric conditioning with either maximal isometric strength (CPIT) or reactive agility (CPRAT). Our findings support the divergent effect hypothesis by demonstrating that CPRAT integration greatly improves T-half and RCOD, two factors considered crucial for handball success. The development of agility requires a specific neuromuscular interaction that incorporates the cognitive and perceptual-motor elements present in reactive agility tasks.

On the other hand, the CPIT has been shown to be the most effective stimulus for increasing lower-limb muscle hypertrophy and maximal voluntary isometric contraction (MVIC). The concept of specificity is highlighted by this adaptive discrepancy: high-intensity isometric training is most appropriate for focusing on the neuronal and structural adaptations of peak strength, but it demonstrates little functional transfer to high-velocity, multidirectional dynamic activities like COD.

Thus, there are two practical implications for in-season training:

1. The CPRAT procedure should be selected when the main purpose is to immediately boost agility and COD productivity, which are critical match-performance attributes.
2. When developing lower-limb maximal strength is the primary goal, the CPIT treatment should be applied gradually and periodically, possibly to set the groundwork for future maximal power.

## Data Availability

The raw data supporting the conclusions of this article will be made available by the authors, without undue reservation.

## References

[B1] Allégue H. Turki O. Oranchuk D. J. Khemiri A. Schwesig R. Chelly M. S. (2023). The effect of combined isometric and plyometric training versus contrast strength training on physical performance in male junior handball players. Appl. Sci. 13, 9069. 10.3390/app13169069

[B2] Aloui G. Souhail H. Hayes L. D. Bouhafs E. G. Chelly M. S. Schwesig R. (2021). Effects of combined plyometric and short sprints training on athletic performance of Male U19 soccer players. Front. Psychol. 12, 714016. 10.3389/fpsyg.2021.714016 34603139 PMC8481369

[B3] Asadi A. (2013). Effects of in-season short-term plyometric training on jumping and agility performance of basketball players. Sport Sci. Health 9, 133–137. 10.1007/s11332-013-0159-4

[B4] Asadi A. Arazi H. (2012). Effects of high-intensity plyometric training on dynamic balance, agility, vertical jump and sprint performance in young male basketball players. J. Sport Health Res. 4, 35–44.

[B5] Asadi A. Arazi H. Ramirez-Campillo R. Moran J. Izquierdo M. (2017). Influence of maturation stage on agility performance gains after plyometric training: a systematic review and meta-analysis. J. Strength cond. Res 31, 2609–2617. 10.1519/JSC.0000000000001994 28557853

[B6] Bayios I. A. Anastasopoulou E. M. Sioudris D. S. Boudolos K. D. (2001). Relationship between isokinetic strength of the internal and external shoulder rotators and ball velocity in team handball. J. Sports Med. Phys. Fit. 41, 229–235. 11447367

[B7] Beckett J. R. Schneiker K. T. Wallman K. E. Dawson B. T. Guelfi K. J. (2009). Effects of static stretching on repeated sprint and change of direction performance. Med. Sci. Sports Exerc. 41, 444–450. 10.1249/MSS.0b013e3181867b95 19127179

[B8] Bedoya A. A. Miltenberger M. R. Lopez R. M. (2015). Plyometric training effects on athletic performance in youth soccer athletes: a systematic review. J. Strength Cond. Res. 29, 2351–2360. 10.1519/JSC.0000000000000877 25756326

[B9] Bouteraa I. Negra Y. Shephard R. J. Chelly M. S. (2020). Effects of combined balance and plyometric training on athletic performance in female basketball players. J. Strength Cond. Res. 34, 1967–1973. 10.1519/JSC.0000000000002546 29489714

[B10] Burgess D. J. Naughton G. A. (2010). Talent development in adolescent team sports: a review. Int. J. Sports Physiol. Perform. 5, 103–116. 10.1123/ijspp.5.1.103 20308701

[B11] Chaabene H. Prieske O. Negra Y. Granacher U. (2018). Change of direction speed: toward a strength training approach with accentuated eccentric muscle actions. Sports Med. 48, 1773–1779. 10.1007/s40279-018-0907-3 29594958

[B12] Chaabene H. Negra Y. Moran J. Prieske O. Sammoud S. Ramirez-Campillo R. (2021). Plyometric training improves not only measures of linear speed, power, and change-of-direction speed but also repeated sprint ability in young female handball players. J. Strength Cond. Res. 35, 2230–2235. 10.1519/JSC.0000000000003128 30946268

[B13] Chelly M. S. Hermassi S. Aouadi R. Khalifa R. Van Den Tillaar R. Chamari K. (2011). Match analysis of elite adolescent team handball players. J. Strength Cond. Res. 25, 2410–2417. 10.1519/JSC.0b013e3182030e43 21869627

[B14] Chelly M. S. Hermassi S. Aouadi R. Shephard R. J. (2014). Effects of 8-week in-season plyometric training on upper and lower limb performance of elite adolescent handball players. J. Strength Cond. Res. 28, 1401–1410. 10.1519/JSC.0000000000000279 24149768

[B15] Cherni Y. Hammami M. Jelid M. C. Aloui G. Suzuki K. Shephard R. J. (2020). Neuromuscular adaptations and enhancement of physical performance in female basketball players after 8 weeks of plyometric training. Front. Physiol. 11, 588787. 10.3389/fphys.2020.588787 33584327 PMC7873906

[B16] Chow C. G. Kong Y. H. Wong C. L. (2022). Reactive-agility in touch plays an important role in elite playing level: reliability and validity of a newly developed repeated up-and-down agility test. J. Sports Sci. Med. 21, 413–418. 10.52082/jssm.2022.413 36157398 PMC9459766

[B17] Christou M. Smilios I. Sotiropoulos K. Volaklis K. Pilianidis T. Tokmakidis S. P. (2006). Effects of resistance training on the physical capacities of adolescent soccer players. J. Strength Cond. Res. 20, 783–791. 10.1519/R-17254.1 17194231

[B18] Craig B. W. (2004). What is the scientific basis of speed and agility? Strength Cond. J. 26, 13–14. 10.1519/00126548-200406000-00002

[B19] Dello Iacono A. Martone D. Milic M. Padulo J. (2017). Vertical-vs. horizontal-oriented drop jump training: chronic effects on explosive performances of elite handball players. J. Strength Cond. Res. 31, 921–931. 10.1519/JSC.0000000000001555 27398920

[B20] Di Russo F. Bultrini A. Brunelli S. Delussu A. S. Polidori L. Taddei F. (2010). Benefits of sports participation for executive function in disabled athletes. J. Neurotrauma 27, 2309–2319. 10.1089/neu.2010.1501 20925480 PMC2996817

[B21] Durnin J. V. Womersley J. (1974). Body fat assessed from total body density and its estimation from skinfold thickness: measurements on 481 men and women aged from 16 to 72 years. Br. J. Nutr. 32, 77–97. 10.1079/bjn19740060 4843734

[B22] Faigenbaum A. D. Kraemer W. J. Blimkie C. J. Jeffreys I. Micheli L. J. Nitka M. (2009). Youth resistance training: updated position statement paper from the national strength and conditioning association. J. Strength Cond. Res. 23, S60–S79. 10.1519/JSC.0b013e31819df407 19620931

[B23] Foster C. Florhaug J. A. Franklin J. Gottschall L. Hrovatin L. A. Parker S. (2001). A new approach to monitoring exercise training. J. Strength Cond. Res. 15, 109–115. 11708692

[B24] Franco-Márquez F. Rodríguez-Rosell D. González-Suárez J. M. Pareja-Blanco F. Mora-Custodio R. Yañez-García J. M. (2015). Effects of combined resistance training and plyometrics on physical performance in young soccer players. Int. J. Sports Med. 36, 906–914. 10.1055/s-0035-1548890 26180903

[B25] Glaister M. Howatson G. Pattison J. R. Mcinnes G. (2008). The reliability and validity of fatigue measures during multiple-sprint work: an issue revisited. J. Strength Cond. Res. 22, 1597–1601. 10.1519/JSC.0b013e318181ab80 18714226

[B26] Hahn D. (2011). Lower extremity extension force and electromyography properties as a function of knee angle and their relation to joint torques: implications for strength diagnostics. J. Strength Cond. Res. 25, 1622–1631. 10.1519/JSC.0b013e3181ddfce3 21386725

[B27] Hammami M. Negra Y. Aouadi R. Shephard R. J. Chelly M. S. (2016). Effects of an In-season plyometric training program on repeated change of direction and sprint performance in the junior soccer player. J. Strength Cond. Res. 30, 3312–3320. 10.1519/JSC.0000000000001470 27135476

[B28] Hammami M. Gaamouri N. Shephard R. J. Chelly M. S. (2019). Effects of contrast strength vs. plyometric training on lower-limb explosive performance, ability to change direction and neuromuscular adaptation in soccer players. J. Strength Cond. Res. 33, 2094–2103. 10.1519/JSC.0000000000002425 29351161

[B29] Hammami M. Ramirez-Campillo R. Gaamouri N. Aloui G. Shephard R. J. Chelly M. S. (2019). Effects of a combined Upper- and lower-limb plyometric training program on high-intensity actions in female U14 handball players. Pediatr. Exerc. Sci. 31, 465–472. 10.1123/pes.2018-0278 31310989

[B30] Hannah R. Minshull C. Buckthorpe M. W. Folland J. P. (2012). Explosive neuromuscular performance of males versus females. Exp. Physiol. 97, 618–629. 10.1113/expphysiol.2011.063420 22308163

[B31] Hermassi S. Chelly M. S. Fathloun M. Shephard R. J. (2010). The effect of heavy-vs. moderate-load training on the development of strength, power, and throwing ball velocity in male handball players. J. Strength Cond. Res. 24, 2408–2418. 10.1519/JSC.0b013e3181e58d7c 20706155

[B32] Herrero A. J. De Vicuña O. a.G. Rábago J. C. M. López J. G. (2006). Parámetros de entrenamiento con electroestimulación y efectos crónicos sobre la función muscular (I). Arch. Med. Deporte 23, 455–462.

[B33] Hewett T. E. Myer G. D. Ford K. R. Heidt R. S. Jr. Colosimo A. J. Mclean S. G. (2005). Biomechanical measures of neuromuscular control and valgus loading of the knee predict anterior cruciate ligament injury risk in female athletes: a prospective study. Am. J. Sports Med. 33, 492–501. 10.1177/0363546504269591 15722287

[B34] Hopkins W. G. Marshall S. W. Batterham A. M. Hanin J. (2009). Progressive statistics for studies in sports medicine and exercise science. Med. Sci. Sports Exerc. 41, 3–13. 10.1249/MSS.0b013e31818cb278 19092709

[B35] Horníková H. Jeleň M. Zemková E. (2021). Determinants of reactive agility in tests with different demands on sensory and motor components in handball players. Appl. Sci. 11, 6531. 10.3390/app11146531

[B36] Hunter S. K. (2014). Sex differences in human fatigability: mechanisms and insight to physiological responses. Acta Physiol. (Oxf) 210, 768–789. 10.1111/apha.12234 24433272 PMC4111134

[B37] Idrizovic K. Gjinovci B. Sekulic D. Uljevic O. João P. V. Spasic M. (2018). The effects of 3-Month skill-based and plyometric conditioning on fitness parameters in junior female volleyball players. Pediatr. Exerc. Sci. 30, 353–363. 10.1123/pes.2017-0178 29478378

[B38] Johnston P. T. Feller J. A. Mcclelland J. A. Webster K. E. (2022). Knee strength deficits following anterior cruciate ligament reconstruction differ between quadriceps and hamstring tendon autografts. Knee Surg. Sports Traumatol. Arthrosc. 30, 1300–1310. 10.1007/s00167-021-06565-0 33876272

[B39] Karcher C. Buchheit M. (2014). On-court demands of elite handball, with special reference to playing positions. Sports Med. 44, 797–814. 10.1007/s40279-014-0164-z 24682948

[B40] Kotzamanidis C. (2006). Effect of plyometric training on running performance and vertical jumping in prepubertal boys. J. Strength Cond. Res. 20, 441–445. 10.1519/R-16194.1 16686577

[B41] Lockie R. G. Jeffriess M. D. Mcgann T. S. Callaghan S. J. Schultz A. B. (2014). Planned and reactive agility performance in semiprofessional and amateur basketball players. Int. J. Sports Physiol. Perform. 9, 766–771. 10.1123/ijspp.2013-0324 24231129

[B42] Lum D. Barbosa T. M. (2019). Brief review: effects of isometric strength training on strength and dynamic performance. Int. J. Sports Med. 40, 363–375. 10.1055/a-0863-4539 30943568

[B43] Luteberget L. S. Spencer M. (2017). High-intensity events in international women's team handball matches. Int. J. Sports Physiol. Perform. 12, 56–61. 10.1123/ijspp.2015-0641 27071136

[B44] Luteberget L. S. Trollerud H. P. Spencer M. (2018). Physical demands of game-based training drills in women's team handball. J. Sports Sci. 36, 592–598. 10.1080/02640414.2017.1325964 28508705

[B45] Maio Alves J. M. Rebelo A. N. Abrantes C. Sampaio J. (2010). Short-term effects of complex and contrast training in soccer players' vertical jump, sprint, and agility abilities. J. Strength Cond. Res. 24, 936–941. 10.1519/JSC.0b013e3181c7c5fd 20300035

[B46] Manchado C. Tortosa-Martínez J. Vila H. Ferragut C. Platen P. (2013). Performance factors in women's team handball: physical and physiological aspects--a review. J. Strength Cond. Res. 27, 1708–1719. 10.1519/JSC.0b013e3182891535 23439330

[B47] Markovic G. (2007). Does plyometric training improve vertical jump height? A meta-analytical review. Br. J. Sports Med. 41, 349–355. 10.1136/bjsm.2007.035113 17347316 PMC2465309

[B48] Markovic G. Mikulic P. (2010). Neuro-musculoskeletal and performance adaptations to lower-extremity plyometric training. Sports Med. 40, 859–895. 10.2165/11318370-000000000-00000 20836583

[B49] Markovic G. Jukic I. Milanovic D. Metikos D. (2007). Effects of sprint and plyometric training on muscle function and athletic performance. J. Strength Cond. Res. 21, 543–549. 10.1519/R-19535.1 17530960

[B50] Matavulj D. Kukolj M. Ugarkovic D. Tihanyi J. Jaric S. (2001). Effects of plyometric training on jumping performance in junior basketball players. J. Sports Med. Phys. Fit. 41, 159–164. 11447356

[B51] Meylan C. Malatesta D. (2009). Effects of in-season plyometric training within soccer practice on explosive actions of young players. J. Strength Cond. Res. 23, 2605–2613. 10.1519/JSC.0b013e3181b1f330 19910813

[B52] Michalsik L. B. Madsen K. Aagaard P. (2015). Physiological capacity and physical testing in male elite team handball. J. Sports Med. Phys. Fit. 55, 415–429. 24402441

[B53] Miller M. G. Herniman J. J. Ricard M. D. Cheatham C. C. Michael T. J. (2006). The effects of a 6-week plyometric training program on agility. J. Sports Sci. Med. 5, 459–465. 24353464 PMC3842147

[B54] Mirwald R. L. Baxter-Jones A. D. Bailey D. A. Beunen G. P. (2002). An assessment of maturity from anthropometric measurements. Med. Sci. Sports Exerc. 34, 689–694. 10.1097/00005768-200204000-00020 11932580

[B55] Myer G. D. Ford K. R. Barber Foss K. D. Liu C. Nick T. G. Hewett T. E. (2009). The relationship of hamstrings and quadriceps strength to anterior cruciate ligament injury in female athletes. Clin. J. Sport Med. 19, 3–8. 10.1097/JSM.0b013e318190bddb 19124976 PMC9928500

[B56] Oliver J. L. Meyers R. W. (2009). Reliability and generality of measures of acceleration, planned agility, and reactive agility. Int. J. Sports Physiol. Perform. 4, 345–354. 10.1123/ijspp.4.3.345 19953822

[B57] Oranchuk D. J. Storey A. G. Nelson A. R. Cronin J. B. (2019). Isometric training and long-term adaptations: effects of muscle length, intensity, and intent: a systematic review. Scand. J. Med. Sci. Sports 29, 484–503. 10.1111/sms.13375 30580468

[B58] Ortega F. B. Lavie C. J. (2018). Introduction and update on obesity and cardiovascular diseases 2018. Prog. Cardiovasc. Dis. 61, 87–88. 10.1016/j.pcad.2018.07.009 29981349

[B59] Pojskic H. Sisic N. Separovic V. Sekulic D. (2018). Association between conditioning capacities and shooting performance in professional basketball players: an analysis of stationary and dynamic shooting skills. J. Strength Cond. Res. 32, 1981–1992. 10.1519/JSC.0000000000002100 29939949

[B60] Radnor J. M. Oliver J. L. Waugh C. M. Myer G. D. Moore I. S. Lloyd R. S. (2018). The influence of growth and maturation on stretch-shortening cycle function in youth. Sports Med. 48, 57–71. 10.1007/s40279-017-0785-0 28900862 PMC5752749

[B61] Ramirez-Campillo R. Garcia-Pinillos F. Chaabene H. Moran J. Behm D. G. Granacher U. (2021). Effects of plyometric jump training on electromyographic activity and its relationship to strength and jump performance in healthy trained and untrained populations: a systematic review of randomized controlled trials. J. Strength Cond. Res. 35, 2053–2065. 10.1519/JSC.0000000000004056 34027912

[B62] Ramirez-Campillo R. García-Hermoso A. Moran J. Chaabene H. Negra Y. Scanlan A. T. (2022). The effects of plyometric jump training on physical fitness attributes in basketball players: a meta-analysis. J. Sport Health Sci. 11, 656–670. 10.1016/j.jshs.2020.12.005 33359798 PMC9729929

[B63] Sassi R. H. Dardouri W. Yahmed M. H. Gmada N. Mahfoudhi M. E. Gharbi Z. (2009). Relative and absolute reliability of a modified agility T-test and its relationship with vertical jump and straight sprint. J. Strength Cond. Res. 23, 1644–1651. 10.1519/JSC.0b013e3181b425d2 19675502

[B64] Scanlan A. Humphries B. Tucker P. S. Dalbo V. (2014). The influence of physical and cognitive factors on reactive agility performance in men basketball players. J. Sports Sci. 32, 367–374. 10.1080/02640414.2013.825730 24015713

[B65] Schabort E. J. Hawley J. A. Hopkins W. G. Mujika I. Noakes T. D. (1998). A new reliable laboratory test of endurance performance for road cyclists. Med. Sci. Sports Exerc. 30, 1744–1750. 10.1097/00005768-199812000-00014 9861609

[B66] Sekulic D. Spasic M. Mirkov D. Cavar M. Sattler T. (2013). Gender-specific influences of balance, speed, and power on agility performance. J. Strength Cond. Res. 27, 802–811. 10.1519/JSC.0b013e31825c2cb0 22580982

[B67] Sekulic D. Krolo A. Spasic M. Uljevic O. Peric M. (2014). The development of a new stop'n'go reactive-agility test. J. Strength Cond. Res. 28, 3306–3312. 10.1519/JSC.0000000000000515 24787675

[B68] Shephard R. J. Bouhlel E. Vandewalle H. Monod H. (1988). Muscle mass as a factor limiting physical work. J. Appl. Physiol. (1985) 64, 1472–1479. 10.1152/jappl.1988.64.4.1472 3378982

[B69] Sheppard J. M. Young W. B. (2006). Agility literature review: classifications, training and testing. J. Sports Sci. 24, 919–932. 10.1080/02640410500457109 16882626

[B70] Slimani M. Chamari K. Miarka B. Del Vecchio F. B. Chéour F. (2016). Effects of plyometric training on physical fitness in team sport athletes: a systematic review. J. Hum. Kinet. 53, 231–247. 10.1515/hukin-2016-0026 28149427 PMC5260592

[B71] Spasic M. Krolo A. Zenic N. Delextrat A. Sekulic D. (2015). Reactive agility performance in handball; development and evaluation of a sport-specific measurement protocol. J. Sports Sci. Med. 14, 501–506. 26336335 PMC4541112

[B72] Spiteri T. Nimphius S. Hart N. H. Specos C. Sheppard J. M. Newton R. U. (2014). Contribution of strength characteristics to change of direction and agility performance in female basketball athletes. J. Strength Cond. Res. 28, 2415–2423. 10.1519/JSC.0000000000000547 24875426

[B73] Suchomel T. J. Nimphius S. Bellon C. R. Stone M. H. (2018). The importance of muscular strength: training considerations. Sports Med. 48, 765–785. 10.1007/s40279-018-0862-z 29372481

[B74] Tillin N. A. Folland J. P. (2014). Maximal and explosive strength training elicit distinct neuromuscular adaptations, specific to the training stimulus. Eur. J. Appl. Physiol. 114, 365–374. 10.1007/s00421-013-2781-x 24292019

[B75] Vincent W. (1995). Statistics in Kinesiology. Champaign, IL, USA: Human Kinetics.

[B76] Vissing K. Brink M. Lønbro S. Sørensen H. Overgaard K. Danborg K. (2008). Muscle adaptations to plyometric vs. resistance training in untrained young men. J. Strength Cond. Res. 22, 1799–1810. 10.1519/JSC.0b013e318185f673 18978625

[B77] Wagner H. Finkenzeller T. Würth S. Von Duvillard S. P. (2014). Individual and team performance in team-handball: a review. J. Sports Sci. Med. 13, 808–816. 25435773 PMC4234950

[B78] Wong Del P. Chan G. S. Smith A. W. (2012). Repeated-sprint and change-of-direction abilities in physically active individuals and soccer players: training and testing implications. J. Strength Cond. Res. 26, 2324–2330. 10.1519/JSC.0b013e31823daeab 22067248

